# Are high flow arteriovenous accesses associated with worse haemodialysis?

**DOI:** 10.1590/2175-8239-JBN-3875

**Published:** 2018-05-28

**Authors:** Ivo Laranjinha, Patrícia Matias, Ana Azevedo, David Navarro, Carina Ferreira, Tiago Amaral, Marco Mendes, Inês Aires, Cristina Jorge, Célia Gil, Anibal Ferreira

**Affiliations:** 1 Dialverca - Clínica de diálise, Forte da Casa, Portugal.; 2 Faculdade de Ciências Médicas, Lisbon, Portugal.; 3 Nephrocare - Clínica de diálise, Vila Franca de Xira, Portugal.; 4 Universidade Nova de Lisboa, Faculdade de Ciências Médicas, Lisboa, Portugal.

**Keywords:** Arteriovenous Fistula, Blood Flow Velocity, Efficiency, Fístula Arteriovenosa, Débito de sangue do acesso, Eficiência

## Abstract

**Introduction:**

An arteriovenous (AV) access flow (Qa) of 400 mL/min is usually sufficient for an effective hemodialysis (HD), but some accesses continue developing and become high flow accesses (HFA). Some authors postulated that an HFA might shift a significant portion of dialyzed blood from the cardiac output, which could decrease HD efficiency and lead to volume overload.

**Objective:**

The aim of our study was to evaluate if HFA is associated with reduced HD efficiency and/or volume overload in prevalent HD patients.

**Methods:**

We performed a 1-year retrospective study and assessed HD efficiency by the percentage of sessions in which the Kt/V > 1.4 and volume overload by bioimpedance spectroscopy.

**Results:**

The study included 304 prevalent HD patients with a mean age of 67.5 years; 62.5% were males, 36.2% were diabetics, with a median HD vintage of 48 months. Sixteen percent of the patients had a HFA (defined as Qa > 2 L/min). In multivariate analysis, patients with HFA presented higher risk of volume overload (OR = 2.67, 95%CI = 1.06-6.71) and severe volume overload (OR = 4.06, 95%CI = 1.01-16.39) and attained dry weight less frequently (OR = 0.37, 95%CI = 0.14-0.94). However, HFA was not associated with lower Kt/V.

**Conclusion:**

Our results suggest that patients with HFA have higher risk of volume overload. However, contrarily to what has been postulated, HFA was not associated with less efficient dialysis, measured by Kt/V. Randomized controlled trials are needed to clarify these questions.

## RESUMO

**Introdução:**

Um débito de sangue de acesso arteriovenoso (AV) (Qa) de 400 mL/min é geralmente suficiente para uma hemodiálise (HD) eficaz, mas alguns acessos continuam se desenvolvendo e se tornam acessos de alto débito (AAD). Alguns autores postularam que um AAD poderia desviar uma porção significativa do sangue dialisado do débito cardíaco, o que poderia diminuir a eficiência da HD e levar à sobrecarga de volume.

**Objetivo:**

O objetivo do nosso estudo foi avaliar se o AAD está associado à redução da eficiência da HD e/ou à sobrecarga de volume em pacientes prevalentes em HD.

**Métodos:**

Foi realizado um estudo retrospectivo de 1 ano, e avaliada a eficiência da HD pela porcentagem de sessões em que o Kt/V > 1,4 e a sobrecarga de volume avaliada pela bioimpedância.

**Resultados:**

O estudo incluiu 304 pacientes prevalentes em HD, com média de idade de 67,5 anos; 62,5% eram do sexo masculino; 36,2% eram diabéticos, com uma mediana de tempo em HD de 48 meses. Dezesseis por cento dos pacientes apresentavam AAD (definida como Qa > 2 L/min). Na análise multivariada, os pacientes com AAD apresentaram maior risco de sobrecarga de volume (OR = 2,67; IC95% = 1,06-6,71) e sobrecarga severa de volume (OR = 4,06; IC95% = 1,01-16,39) e atingiram o peso seco com menor frequência (OR = 0,37, IC 95% = 0,14-0,94). No entanto, o AAD não foi associado uma menor razão Kt/V.

**Conclusão:**

Nossos resultados sugerem que pacientes com AAD apresentam maior risco de sobrecarga de volume. No entanto, ao contrário do que foi postulado, o AAD não foi associado à diálise menos eficiente, medida pelo Kt/V. Ensaios clínicos randomizados são necessários para esclarecer essas questões.

## INTRODUCTION

Adequate hemodialysis (HD) requires a functional vascular access that is able to deliver a flow rate of at least 350-400 mL/min with minimal recirculation for the total duration of treatment.^1^^,^ 2 The monitoring of the access flow (Qa) is very important for the early detection of access dysfunction^3^ such as high flow access (HFA). Although a higher blood flow allows easy needling and excellent blood flow rate to the dialyzer (Qb), it has been related to some systemic deleterious consequences.^4^

There is no exact definition of the Qa level above which a HFA should be considered and no consensual guideline about the ideal or normal access blood flow. The guidelines from the Vascular Access Society defined an arteriovenous fistula (AVF) as a high flow fistula with a Qa between 1-1.5 L/min and a measured cardiopulmonary recirculation (CPR) [Qa/cardiac output (CO)] greater than 20%.^5^ In the absence of an exact description of a HFA, a Qa > 2 L/min is pragmatically used as a cut-off point, since it increases the risk of cardiac failure in HD patients with a Qa/CO > 20-30%.^6^^-^ 8

Considering the structural and functional cardiac adaptations associated with an AV access construction, and especially the higher risk for heart failure in patients with HFA, some patients become pre-load dependent, which causes intra-dialytic hypotension with lower ultrafiltration.^6^^,^ 7 Furthermore, the construction of an AV access leads to the creation of a left to right extracardiac shunt, which causes CPR, i.e., dialyzed blood from the AV access is directed to the right ventricle and pulmonary circulation, is then pumped into the systemic circulation and a portion of this dialyzed blood re-enters the access. The re-entrance of the dialyzed and systemic blood mix into the access could lead to poor solute clearance.^1^^,^ 7 ^,^ 9 This mix decreases the solute concentration between blood and dialysate, which reduces the solute removal from the blood*.* Schneditz *et al.* were the first to show the association between CPR and HD efficiency with a theoretical model.^9^^,^ 10

Maintaining a HFA could cause functional and structural cardiac changes (cardiac toxicity) which could in the end reduce the CO. Considering the heart has a limited capacity to increase the CO, accesses with a higher flow deviate a higher proportion of the CO, i.e. they have a greater CPR. That way, if the same access (with the same Qa - numerator) is maintained and the CO is reduced (as a consequence of the cardiac toxicity of these accesses - denominator), it means that the ratio Qa/CO (percentage of blood deviated from the CO to the access) is high, which means a higher CPR.^9^^-^ 11

Considering these unresolved questions, we performed a study to evaluate if a higher Qa was associated with reduced HD efficiency and/or volume overload in prevalent HD patients. We hypothesized that patients with HFA have a lower tolerability to ultrafiltration, have more volume overload, and could present a high CPR, reducing HD efficiency.

## SUBJECTS AND METHODS

### STUDY DESIGN

This was an observational, 1-year retrospective, single-center study of a cohort of adult prevalent HD patients. The studied population was divided in two groups (HFA and non-HFA) according to the mean value of the last three Qa measurements, separated by at least one month. We defined a HFA as a Qa higher than 2 L/min.

All patients were dialyzed with high flux helixone filters (Fresenius^®^), ultrapure water dialysate (evaluated monthly by kinetic chromogenic test) and online post-dilution hemodiafiltration. We used the dialysis FX CorDiax600 (Fresenius Medical Care), which has an effective surface of 1.6 m^2^ and Intrinsic Clearance for Urea (KoA) of 1.148. All patients included in the study were prescribed 4-hour HD sessions. The median HD vintage was 48 months (IQ range 24-96).

#### CLINICAL CHARACTERISTICS

Patient's data on age, gender, baseline comorbidity (diabetes, hypertension, coronary artery disease, peripheral vascular disease, and cerebrovascular disease), access type, location, and date of angiographic and surgical interventions were all extracted from the clinical database Euclid^®^.

Biochemical parameters such as hemoglobin and albumin (reference value > 4.0 g/dL) were evaluated monthly and mean values were determined. Each patient underwent an echocardiographic examination during the study period (M mode and 2-D) and left ventricular mass index (LVMI) was calculated by the Devereux formula.^12^

Patients with NYHA class greater than II were excluded from the study.

#### ACCESS BLOOD FLOW (QA) MEASUREMENT

Access flow was routinely measured by thermodilution, using the Fresenius Medical Care Blood Temperature Monitor (BTM) at a Qb of 300 mL/min using a twister device. This measurement is always started in the first hour of treatment. In grafts, the Qa was assessed monthly and in fistulas it was assessed depending on the measurement itself: if the last measure was lower than 600 mL/min, the Qa was measured monthly, if it was between 600 and 1000 mL/min, the evaluation was postponed to 4 months, and if the last Qa was higher than 1000 mL/min, the next evaluation was postponed to 1 year.

#### VOLUME STATUS ASSESSMENT

Patient's volume status was evaluated monthly by bioimpedance spectroscopy using the Fresenius Medical Care Body Composition Monitor (BCM).^13^ Both the absolute and relative fluid overload were assessed. According to BCM validation studies, the volume status was classified in three categories:

- Dry weight (absolute fluid overload below 1 L)
- Volume overloaded (absolute fluid overload above 1.1 L)
- Severe volume overloaded (absolute fluid overload above 2.5 L)

#### DELIVERED DIALYSIS DOSE

Kt/V was measured using the Fresenius Medical Care Online Clearance Monitor (OCM) in all HD sessions during 1 year (approximately 139 sessions per patient). For each patient, we calculated the percentage of sessions in which the Kt/V goal (Kt/V > 1.4) was achieved.

### STATISTICAL ANALYSIS

Variables were reported as frequencies for categorical variables, mean values with SD for continuous variables with normal distribution, and median values with interquartile ranges for continuous variables non-normally distributed. We applied Shapiro-Wilk test to evaluate the normality of our data. Comparison between groups (HFA and non-HFA) was performed using *T*-Test for normally distributed variables, Wilcoxon test for non-normally distributed variables and *x* ^2^ test for categorical variables.

For the purpose of the analysis, HFA was used as predictor and volume overload, mean value of Kt/V throughout the year, and % of Kt/V > 1.4 as outcome variables.

The relationship between HFA and volume overload was studied using univariate and multivariate logistic regression models to adjust for potential confounders. The multivariable analysis was adjusted for age (statistically different in the univariable analysis), HD vintage, serum albumin, and LVMI.

The relationship between HFA and Kt/V was studied using the Spearman correlation in a univariate analysis. Multivariate analysis was performed using a linear regression model. These models were adjusted for age (statistically different in the univariate analysis) and for dry weight, pump speed, and time of HD per session (important determinants of HD efficacy).

Statistical analysis was performed with SPSS system 21.0. For all comparisons, a *p* < 0.05 was considered statistically significant.

## RESULTS

### POPULATION

The study included 304 patients, 190 (62.5%) males, with a mean age of 67.5 ± 14.8 years old. A total of 110 (36.2%) patients were diabetic, 219 (72.0%) had hypertension, 90 (29.6%) had coronary artery disease, 70 (23%) peripheral arterial disease and 63 (20.7%) had cerebrovascular disease (Table 1).

**Table 1:** Clinical, vascular access, and hemodialysis parameters of the studied population

Variable	Patients (n = 304)
Age, years	67.5 ± 14.8
Gender, male	190 (62.5)
Race, Caucasian	288 (94.7)
HD vintage, months	48 (24-96)
HD session duration, minutes	245 (245-247)
Blood pump speed (Qb), mL/min	442.7 ± 17.9
Diabetes	110 (36.2)
Hypertension	219 (72.0)
Access type	
Fistula	225 (74.0)
Graft	79 (26.0)
Access location, proximal	193 (63.5)
Access with Qa ≥ 2 L/min	48 (15.8)
Mean Kt/V	1.98 ± 0.39
Dry weight (OH < 1 L)	269 (88.5)
Volume overload (OH > 1 L)	35 (11.5)
Severe volume overload (OH > 2.5 L)	10 (3.3)

* Values reported as mean±SD, median (interquartile range) or frequencies [n (%)].

All patients had a functioning AV access for HD: approximately three quarters (n = 225; 74.0%) had a fistula and one quarter (n = 79, 26%) had a graft. In our population, 15.8% of the patients had a HFA, i.e. an access with Qa > 2 L/min. The mean Kt/V value was 1.98 and 88.5% of the patients achieved their dry weight.

Patients with a HFA were younger (62.1 *vs*. 68.5 years, *p* = 0.015), were under HD for longer time (60 *vs*. 48 months, *p* = 0.034) and had a lower prevalence of diabetes (14.6 *vs*. 40.2%, *p* < 0.001). There was no significant difference in gender distribution and prevalence of coronary artery disease, cerebrovascular disease, and peripheral artery disease between groups (Table 2).

**Table 2:** Clinical and laboratory characteristics of patients with HFA and non-HFA

	Qa < 2 L/min (n = 256)	Qa ≥ 2 L/min (n = 48)	*p*
Age, years	68.5 ± 14.1	62.1 ± 17.5	0.015
Gender, male	155 (60.5)	35 (72.9)	ns
Race, Caucasian	245 (95.7)	43 (89.6)	ns
HD vintage, months	48 (24-84)	60 (27-108)	0.034
HD sessions duration, minutes	245 (245-247)	246 (245-247)	ns
Blood pump speed (Qb), mL/min	442.7 ± 18.0	443.3 ± 17.8	ns
Dry weight (kg)	69.9 ± 13.5	70.7 ± 11.3	ns
Diabetes	103 (40.2)	7 (14.6)	< 0.001
Hemoglobin (g/dL)	11.1 ± 1.1	11.2 ± 1.1	ns
Serum Albumin (g/dL)	4.0 ± 0.3	4.1 ± 0.3	ns
Access type			
Fistula	181 (70.7)	44 (91.7)	0.001
Graft	75 (29.3)	4 (8.3)	
Access location, proximal	157 (61.3)	36 (75.0)	0.043
Pulse pressure, mmHg	78.0 ± 15.9	71.7 ± 17.6	0.037
Mean Blood Pressure, mmHg	92.0 ± 13.2	90.0 ± 14.5	ns
Heart Rate, bpm	71.7 ± 10.6	72.1 ± 10.0	ns
Left Ventricular Mass Index (g/m^2^)	130.2 ± 30.9	146.2 ± 47.3	0.035
Left Ventricular Ejection Fraction (%)	60.7 ± 13.6	55.1 ± 17.8	ns
Pulmonary Artery Pressure (mmHg)	39.2 ± 12.9	34.1 ± 13.3	ns
Hypertension	182 (71.1)	37 (77.1)	ns
Coronary Artery Disease	80 (31.3)	10 (20.8)	ns
Peripheral artery disease	63 (24.6)	7 (14.6)	ns
Cerebrovascular disease	56 (21.9)	7 (14.6)	ns

* Values reported as mean ± SD, median (interquartile range) or frequencies [n (%)].

In respect to access characteristics, we found that Qa ≥ 2 L/min was more frequent in fistulas comparatively to grafts (91.7 *vs*. 8.3%, *p* = 0.001) and in proximal accesses than in distal (75 *vs*. 25%, *p* = 0.043).

Patients with a HFA had higher left ventricular mass index (146.2 *vs*. 130.2 g/m^2^, *p* = 0.035) and lower pulse pressure (71.7 *vs*. 78.0 mmHg, *p* = 0.037). Although not statistically significant, patients with a HFA presented a higher proportion of hypertension (77.1 *vs*. 71.1%) and lower LVEF (55.1 *vs*. 60.7%).

#### Volume overload

Eighty-nine percent of the patients achieved the dry weight and 11.5% had volume overloaded (OH > 1 L).

In univariate analysis, we found that patients with a HFA had severe volume overloaded more frequently (2.3 *vs*. 8.3 %, OR 3.79, 95%CI = 1.03-13.97). However, the distribution of patients for dry weight and volume overload categories was not different between groups.

A logistic regression analysis was performed to verify the effect of having a HFA on the chance of having volume overload adjusting for age (years), HD vintage (months), serum albumin (g/dL), and LVMI (g/m^2^). We found that an access with Qa > 2 L/min was significantly associated with the risk of volume overload (OR = 2.67, 95%CI = 1.06-6.71) and severe volume overload (OR = 4.06, 95%CI = 1.01-16.39). In the same analysis we also found that patients with HFA attained the dry weight less frequently (OR = 0.37, 95%CI = 0.14-0.94).

#### Kt/V

The mean Kt/V of our population was 1.98 and the Kt/V goal was attained in 97% of the sessions. Patients with a HFA did not have a different mean Kt/V value or a different proportion of sessions in which the Kt/V was attained comparing with patients with an AV access of Qa ≤ 2 L/min.

We performed a multiple regression analysis to investigate whether the Kt/V value could be predicted based on Qa > 2 L/min, in a model adjusted to age (years), dry weight (Kg), blood pump speed (mL/min) and time of each HD session (minutes). This model was not significant (Table 3).

**Table 3:** Efficiency and volume status characteristics of patients with HFA and non-HFA (univariate and multivariate analysis)

	Univariate analysis				Multivariate analysis ^§^
	Qa < 2 L/min (n = 256)	Qa ≥ 2 L/min (n = 48)	OR (95% CI)	*p*	OR (95% CI)
Dry weight (OH ≤ 1 L)	230 (89.8)	39 (81.3)	0.49 (0.21-1.12)	ns	0.37 (0.14-0.94)
volume overload (OH > 1 L)	26 (10.2)	9 (18.8)	2.04 (0.89-4.68)	ns	2.67 (1.06-6.71)
Severe volume overload (OH > 2.5 L)	6 (2.3)	4 (8.3)	3.79 (1.03-13.97)	0.056	4.06 (1.01-16.39)
% VO > 15%	52 (22.0)	9 (21.4)	0.965 (0.43-2.15)	ns	0.79 (0.33-1.93)
% VO > 20%	12 (5.1)	3 (7.1)	1.44 (0.39-5.32)	ns	1.65 (0.39-6.99)
Kt/V	1.99 ± 0.40	1.93 ± 0.35	0.52 (0.07-1.18)	ns	0.03 (0.00-3.09)
% of sessions that Kt/V was attained in 1 year	97.2	95.3	1.69 (0.34-8.40)	ns	1.33 (0.20-8.71)

* Values expressed as mean±SD, median or frequencies [n (%)]. Relative pre-dialytic volume overload (% VO) was calculated as: % VO = VO [L]/ extracellular water [L]*100 at baseline.

§ Multivariate analysis: logistic regression for binary outcomes and linear regression for continuous outcomes. The models were adjusted for age (years), dry weight (kg), pump speed (mL/min), and HD sessions length (minutes) to study the HD efficiency (Kt/V) and for age, HD vintage, serum albumin (g/dL) and LVMI to study the volume status.

We also compared the patients with Qa < 1 L/min with patients with Qa > 1 L/min and the results were not different from those described above.

## DISCUSSION

We believe this is the first study that investigated the relationship between HFA, volume overload, and HD efficiency. We found that having a HFA, at least when the Qa was greater than 2 L/min, was associated with a higher risk of volume overload, but not to dialysis efficiency, evaluated by Kt/V.

There are many studies about the immediate and late cardiac and neurohormonal adaptations after the creation of an AV access. These modifications lead to a compensatory increase in CO, heart rate, cardiac contractility, and blood volume.^10^^,^ 14 Some studies about the cardiac hemodynamic effects of AV accesses suggest that patients with a HFA have elevated CO, higher cardiac index, less subendocardial perfusion, and increased left ventricular cavity size.^5^^,^ 14 ^-^ 16 These patients could also present more cardiac diastolic dysfunction and higher ANP/BNP levels.^10^

Several cases of high-output cardiac failure in patients with a HFA have been reported,^17^^-^ 19 especially in transplanted patients. A Qa/CO ratio higher than 0.3 is considered a risk factor for high-output cardiac failure, however this cut-off has not yet been validated in prospective trials.^20^ Other studies showed echocardiographic and clinical improvement following AV access closure or flow reduction.^21^^-^ 25 An established relationship between Qa and CO was described by Basile *et.al*.^6^ in a 3^rd^ order polynominal regression model, in which the CO did not vary significantly for Qa between 0.95 and 2.2 L/min. These authors also show that a Qa > 2 L/min was a strong predictor of high-output heart failure.^6^

Prospective studies found indirect signs (e.g. increased serum ANP) that the creation of an AV access develops a state of volume overload, at least immediately after the creation,^10^^,^ 26 which have been demonstrated in traumatic fistulas. Besides the increase in blood volume, it was also demonstrated that in traumatic fistulas the greater the fistula blood flow the greater the increase in blood volume.^27^

In our cohort study, patients with a HFA presented higher LVMI, and although not statistically significant, these patients also had a higher proportion of hypertension and lower LVEF.

The cardiac adaptations in patients with a HFA could be associated with a lower tolerability to ultrafiltration and interfere with the achievement of dry weight, which could have prognostic consequences.^7^ In this study, we hypothesized that a higher Qa is associated with a higher risk of volume overload and our findings support this hypothesis. The odds ratio of volume overload and severe volume overload were 2.67 and 4.06 times higher for patients with a HFA than for patients with Qa < 2L/min. We also found that the patients with a HFA attained dry weight less frequently, with an odds ratio 0.37 times lower compared to patients with Qa < 2 L/min.

The making of an AV access leads to a left to right extra-cardiac shunt causing CPR, and a significant proportion of dialyzed blood re-enters the access, which could theoretically lead to poor solute clearance. Thus, accesses with higher blood flow drive a higher percentage of CO to the access, i.e., have greater CPR. Some authors postulated that a persistent HFA could be associated with under-dialysis.^8^ These authors reinforce the important potential for cardiac toxicity, but also the potential systemic consequences of a HFA, such as the "global steal syndrome", under-dialysis, and wasting.^7^^,^ 27

Contrarily to what has been postulated, the higher CPR related with a HFA is not associated with lower HD efficiency. We should note that HD adequacy has multiple components, such as nutrition, anemia management, and mineral and bone disorders. Therefore, this issue needs to be clarified in prospective randomized studies, where other components of HD efficiency are considered, besides the Kt/V.

Although a previous study did not find an increased risk of all-cause mortality associated with higher Qa,^28^ studies are needed to investigate if, when, and which patients would benefit from the reduction of Qa in HFA.^29^

Our results corroborate the new "patient first" paradigm currently accepted in the field of vascular accesses for hemodialysis in opposition to the old "fistula first" paradigm. This change was based on data suggesting that the presence of an AVF can contribute to the high CV morbidity in HD patients, called by Richard Amerling "arteriovenous fistula toxicity".^7^ With this new paradigm, experts want to alert that AVF is not the best choice for all patients. The best vascular access for each patient must be defined individually, based on the risk of heart failure and/or ischemic steal syndrome, CV comorbidities, patient desires, life expectancy, etc.^7^^,^ 8

A limitation of our study is its retrospective nature and an HD population from a single center, which could interfere in the generalization of our results. Another important limitation is that CO, which is a very important determinant of Qa, was not evaluated in our study, as echocardiography routine performed in our center does not include doppler study.

In conclusion, our results suggest that a HFA is associated with higher risk of volume overload and lower capacity of reach dry weight. Contrarily to what has been postulated, patients with a HFA do not have less efficient HD, measured by Kt/V. Randomized controlled trials are needed to examine whether higher Qa adds difficulty in reaching dry weight, if it is cardiovascular risk factor, and if it is associated with under dialysis.

## References

[1] Sidawy AN, Gray R, Besarab A, Henry M, Ascher E, Silva M (2002). Recommended standards for reports dealing with arteriovenous
hemodialysis accesses. J Vasc Surg.

[2] McCarley P, Wingard RL, Shyr Y, Pettus W, Hakim RM, Ikizler TA (2001). Vascular access blood flow monitoring reduces access morbidity
and costs. Kidney Int.

[3] Wijnen E, Planken N, Keuter X, Kooman JP, Tordoir JH, de Haan MW (2006). Impact of a quality improvement programme based on vascular
access flow monitoring on costs, access occlusion and access
failure. Nephrol Dial Transplant.

[4] Agarwal AK (2015). Systemic Effects of Hemodialysis Access. Adv Chronic Kidney Dis.

[5] Wijnen E, Keuter XH, Planken NR, van der Sande FM, Tordoir JH, Leunissen KM (2005). The relation between vascular access flow and different types of
vascular access with systemic hemodynamics in hemodialysis
patients. Artif Organs.

[6] Basile C, Lomonte C, Vernaglione L, Casucci F, Antonelli M, Losurdo N (2008). The relationship between the flow of arteriovenous fistula and
cardiac output in haemodialysis patients. Nephrol Dial Transplant.

[7] Amerling R, Ronco C, Kuhlman M, Winchester JF (2011). Arteriovenous fistula toxicity. Blood Purif.

[8] Basile C, Lomonte C (2012). Pro: the arteriovenous fistula is a blessing of
God. Nephrol Dial Transplant.

[9] Schneditz D, Kaufman AM, Polaschegg HD, Levin NW, Daugirdas JT (1992). Cardiopulmonary recirculation during hemodialysis. Kidney Int.

[10] Iwashima Y, Horio T, Takami Y, Inenaga T, Nishikimi T, Takishita S (2002). Effects of the creation of arteriovenous fistula for hemodialysis
on cardiac function and natriuretic peptide levels in CRF. Am J Kidney Dis.

[11] Miller GA, Hwang WW (2012). Challenges and management of high-flow arteriovenous
Fistulae. Semin Nephrol.

[12] Devereux RB, Alonso DR, Lutas EM, Gottlieb GJ, Campo E, Sachs I (1986). Echocardiographic assessment of left ventricular hypertrophy:
comparison to necropsy findings. Am J Cardiol.

[13] Ponce P, Pinto B (2011). Measuring vascular access flow: the accuracy of different
methods. Port J Nephrol Hypert.

[14] Savage MT, Ferro CJ, Sassano A, Tomson CR V (2002). The impact of arteriovenous fistula formation on central
hemodynamic pressures in chronic renal failure patients: a prospective
study. Am J Kidney Dis.

[15] Dundon BK, Torpey K, Nelson AJ, Wong DT, Duncan RF, Meredith IT (2014). The deleterious effects of arteriovenous fistula-creation on the
cardiovascular system: a longitudinal magnetic resonance imaging
study. Int J Nephrol Renovasc Dis.

[16] Ori Y, Korzets A, Katz M, Erman A, Weinstein T, Malachi T (2002). The contribution of an arteriovenous access for hemodialysis to
left ventricular hypertrophy. Am J Kidney Dis.

[17] Stern AB, Klemmer PJ (2011). High-output heart failure secondary to arteriovenous
fistula. Hemodial Int.

[18] Khreiss M, Haddad FF, Musallam KM, Medawar W, Daouk M, Khalil I (2009). High-output cardiac failure secondary to a large arteriovenous
fistula: a persistent threat to the dialysis and kidney transplant
patient. NDT Plus.

[19] Raza F, Alkhouli M, Rogers F, Vaidya A, Forfia P (2015). Case series of 5 patients with end-stage renal disease with
reversible dyspnea, heart failure, and pulmonary hypertension related to
arteriovenous dialysis access. Pulm Circ.

[20] MacRae JM, Pandeya S, Humen DP, Krivitski N, Lindsay RM (2004). Arteriovenous fistula-associated high-output cardiac failure: a
review of mechanisms. Am J Kidney Dis.

[21] Unger P, Wissing KM, de Pauw L, Neubauer J, Borne P (2002). Reduction of left ventricular diameter and mass after surgical
arteriovenous fistula closure in renal transplant recipients. Transplantation.

[22] van Duijnhoven EC, Cheriex EC, Tordoir JH, Kooman JP, van Hooff JP (2001). Effect of closure of the arteriovenous fistula on left
ventricular dimensions in renal transplant patients. Nephrol Dial Transplant.

[23] Dundon BK, Torpey DK, Nelson AJ, Wong DTL, Duncan RF, Meredith IT (2014). Beneficial cardiovascular remodeling following arterio-venous
fistula ligation post-renal transplantation: a longitudinal magnetic
resonance imaging study. Clin Transplant.

[24] Balamuthusamy S, Jalandhara N, Subramanian A, Mohanaselvan A (2016). Flow reduction in high-flow arteriovenous fistulas improve
cardiovascular parameters and decreases need for
hospitalization. Hemodial Int.

[25] Murray BM, Rajczak S, Herman A, Leary D (2004). Effect of surgical banding of a high-flow fistula on access flow
and cardiac output: intraoperative and long-term
measurements. Am J Kidney Dis.

[26] Ori Y, Korzets A, Katz M, Perek Y, Zahavi I, Gafter U (1996). Haemodialysis arteriovenous access--a prospective haemodynamic
evaluation. Nephrol Dial Transplant.

[27] Alkhouli M, Sandhu P, Boobes K, Hatahet K, Raza F, Boobes Y (2015). Cardiac complications of arteriovenous fistulas in patients with
end-stage renal disease. Nefrologia.

[28] Al-Ghonaim M, Manns BJ, Hirsch DJ, Gao Z, Tonelli M (2008). Relation between access blood flow and mortality in chronic
hemodialysis patients. Clin J Am Soc Nephrol.

[29] Basile C, Lomonte C (2011). When and how should an arterio-venous access be modified because
of a high blood flow rate?. Semin Dial.

